# Kinematic Features of Mandibular Movement during Mastication in Geriatric Individuals Who Are Provided with a Dysphagia Diet at Long-Term Care Facilities

**DOI:** 10.3390/nu15102273

**Published:** 2023-05-11

**Authors:** Enri Nakayama, Haruka Tohara, Mayu Sakai, Masato Iida, Kimiko Abe, Koichiro Ueda

**Affiliations:** 1 The Department of Dysphagia Rehabilitation, Nihon University School of Dentistry, Tokyo 101-8310, Japan; 2 Department of Dysphagia Rehabilitation, Division of Gerontology and Gerodontology, Tokyo Medical and Dental University, Tokyo 113-8549, Japan

**Keywords:** mastication, diet, geriatrics, long-term care facility

## Abstract

Providing a normal diet to a care recipient who is unable to form an adequate bolus may cause suffocation or aspiration pneumonia. We investigated whether differences in kinematic data of mandibular movements during mastication can be used as an indicator of the need for a dysphagia diet in the elderly in long-term care facilities. We included 63 participants who were provided with solid food in two long-term care facilities. The primary outcome variable was the kinematic data on mandibular movement during cracker chewing. The analysis results were compared between the normal and dysphagia diet groups. Logistic regression analysis and receiver operating characteristic curve analyses were performed. Significant differences were observed in the masticatory time, cycle frequency, total change amount, number of linear motions, and circular motion frequency between the normal and modified diet groups. The odds ratio for the circular motion frequency was −0.307, and the calculated cutoff value was 63%, with a sensitivity of 71.4%, a specificity of 73.5%, and an area under the curve of 0.714. Thus, these characteristics may be useful for detecting care recipients who need to be provided with a dysphagia diet. Moreover, the circular motion frequency could be used as a screening test to identify people who need a dysphagia diet.

## 1. Introduction

Providing facility residents with a diet that does not match their masticatory ability may cause choking [1] and aspiration pneumonia [2]. The masticatory ability of facility residents gradually declines because of the effects of dementia [3], sarcopenia [4], and tooth loss [5], among other factors. Since these progressions are not drastic, we sometimes see cases where there is a mismatch between their masticatory function and the food provided, without the caregiver noticing it. Therefore, it is necessary to develop a test that allows caregivers to easily check whether a nursing home resident’s diet suits their masticatory ability.

One non-invasive and simple method of evaluating masticatory function uses gummy jelly [6] or gum [7]. However, these methods require that they be spit out of the mouth at a specified time. Participants with cognitive decline who cannot behave as instructed may swallow them without exhaling them. Moreover, participants with reduced chewing abilities and at risk of swallowing without chewing are ethically problematic as they may inhale or choke on gummies and gums.

In recent years, the Test of Masticating and Swallowing Solids (TOMASS) has been introduced as a method to measure chewing time and count the number of masticatory cycles and swallows by observing the movement of the mandible and thyroid cartilage while the subject is ingesting crackers [8]. TOMASS has reported utility in both healthy participants and patients with dysphagia [9]. In addition, the Saku-Saku test is used as a method to evaluate masticatory function by observing mandible movements during mastication [10]. In this method, subjects are divided into those with good mandibular rotation and those with poor mandibular rotation based on the movement of the mandible while eating rice crackers. Good mandibular rotation was defined when the mandible opens centrally or slightly towards the chewing side and closes lateral to the opening path, whereas simple linear movements opening and closing along a similar path were defined as poor mandibular rotation. This method shows good reliability between examiners and reports, and it has been demonstrated that it is useful for assessing masticatory function in dysphagic patients. However, whether this test can be used to detect people who need to be provided with a dysphagia diet has not been investigated. Therefore, we performed the Saku-Saku test on residents of nursing care facilities provided with a dysphagia diet as a pilot study. However, because most of the subjects were observed to have both types of mandibular rotation, we could not classify them as having good or bad mandibular rotation, as performed in the previous study. Based on the results of this pilot study, we thought that the Saku-Saku test could be effective in detecting individuals with obvious masticatory dysfunction who have difficulty ingesting solid food but was not suitable for detecting people who need to be provided with a dysphagia diet. However, the Saku-Saku test is an excellent examination method that can be performed by anyone without special skills, so we planned to develop an indicator for detecting those who need dysphagia diets based on this test. For that purpose, it is necessary to clarify the characteristics of the masticatory movements of people who are provided with a dysphagia diet.

The purpose of this study was to develop a test of masticatory ability that could determine whether a dysphagia diet should be provided. First, we investigated kinematic data, such as the masticatory movement pathways of geriatric individuals in long-term care facilities, and examined items associated with the provision of a dysphagia diet. Next, we examined the effective indicators for detecting those who need to be provided with a dysphagia diet from among the survey items. We believe that the results of this study will provide an opportunity to review the dietary forms offered to facility residents and contribute to reducing the risk of aspiration and suffocation.

## 2. Materials and Methods

### 2.1. Participants

This was a cross-sectional study, and it was conducted at two facilities in Japan between 2020 and 2021. At these facilities, nutritionists check every month whether there are any problems with the meals of the residents. If there is a resident who has dietary problems, speech pathologists, nurses, and registered dietitians consult with each other to adjust their diet and environment. If any problems persist, residents will undergo a video endoscopy of swallowing by a dentist and receive advice on an optimal diet. The participants were older people who require assistance in daily life due to decreased physical and cognitive functions. Patients who could orally consume solid food and consented to this study were included. Exclusion criteria included: (1) an inability to fulfill simple requests due to cognitive decline; (2) an inability to eat while sitting on a chair; (3) the low endurance of a short survey; (4) no occlusal contact with molars (including dentures); and (5) a history of oral cancer, dental disorders causing painful mastication, or defective dentures. The nurses in the facilities selected the participants, and all the participants or their families received a verbal and written explanation of this study and provided written informed consent. The Institutional Review Board approved this study (Approval No. EP19D015-1), and it was conducted in accordance with the Declaration of Helsinki.

### 2.2. Observation of Masticatory Motion

**Figure 1:**
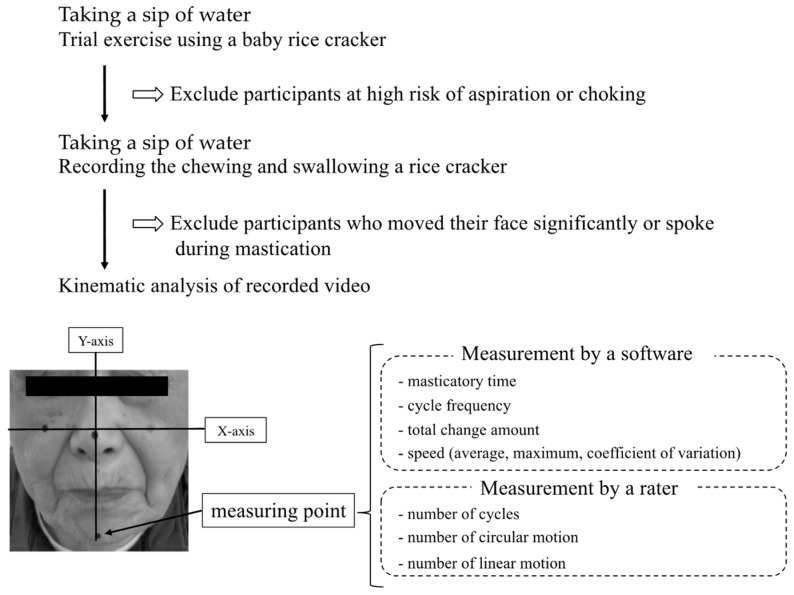
The experimental sequence.

Color stickers (5 mm diameter) were attached to the most protruded parts of the cheekbones, the apex of the nose, and the tip of the participant’s mandible, and a throat microphone (Inkou mike; NZ-210CjK, NANZU, Shizuoka, Japan) was also attached approximately 2 cm outside the laryngeal prominence of the thyroid cartilage. This microphone recorded swallowing sounds caused by the passage of the bolus through the pharynx [11]. The participants were in a wheelchair or armchair. First, after they took a sip of water with the viscosity adjusted as usual, the participants were given one-third of a baby rice cracker (Hai Hain^®^ Baby rice cracker, Kameda Seika Co., Ltd., Niigata, Japan) that became muddy after chewing as a trial exercise. Based on observations during this trial exercise, participants were excluded if they were judged to be at high risk of aspiration or choking during the subsequent procedures. Subsequently, after they took a sip of water again, they were given a 2 g rice cracker (Happy Turn^®^ soft rice cracker, Kameda Seika Co., Ltd., Niigata, Japan) [10] and instructed to keep their faces still while looking at the camera and chewing. We held the iPad (iPad Pro, Apple Inc., Cupertino, CA, USA) directly in front of the participant’s face and recorded mandibular movements along with the corresponding swallowing sounds from the throat microphone. However, participants were excluded from the study if they moved their faces so much that they were not suitable for image analysis or if they spoke during mastication (Figure 1).

### 2.3. Outcome Measures

The recorded images were analyzed using image analysis software (DIPP-Motion PRO 2D; DITECT Corporation, Tokyo, Japan). Among the images of the participants chewing the rice cracker, the masticatory movement from when the first crushing sound was heard to when the first swallowing sound was heard was analyzed. The line passing through the stickers attached to the left and right cheekbones was the *x*-axis; the line passing through the center of the sticker attached to the apex of the nose, and the line perpendicular to the *x*-axis was the *y*-axis. Subsequently, we measured the movement of the sticker attached to the tip of the lower jaw. In addition, the period from the start of the mandible descent to the return to the original position was defined as one cycle. The measurements included: the time from starting mastication to swallowing (masticatory time), the total number of cycles (number of cycles), the cycle frequency (the number of cycles/mastication time), the total change amount, average speed, maximum speed, and speed coefficient of variation. Furthermore, we counted the number of times by classification into the following types for each cycle according to the locus of the mandible. The circular motion was defined when the mandible opens centrally or slightly towards the chewing side and closes lateral to the opening path [10]. A simple linear movement with opening and closing along a similar path was defined as a linear motion [10]. The number of each motion was counted, and the circular motion frequency (number of circular motions/total number of cycles) was calculated. These counts were made by a dentist specializing in dysphagia using a slow-motion replay of recorded footage in which the chin marker trajectories were displayed (Figure 1). Calculating the number of samples required to compute the intraclass correlation coefficient (ICC) in this measurement method with an assumed ICC of 0.8, two observations, a significance level of 0.05, and a power of 0.8, the sample size was 8 cases. Therefore, calculating the ICC (1.1) of the circular and linear motion using the 10 subjects initially collected yields 0.963 and 0.968, respectively; thus, it was highly reliable. In addition, the interrater reliability was confirmed by two dentists who were different from this study’s rater. In these results, the ICCs (2.1) for circular and linear motions were 0.871 and 0.850, respectively; thus, the method had minimal variation among the evaluators. Based on these results, we decided to adopt this method in this study.

### 2.4. Other Measurements

Nurses and dietitians in the long-term care facilities surveyed the following items immediately before the survey. The survey items were age, sex, history of cerebrovascular disease, activities of daily living (ADL), body mass index (BMI), and nutritional status. ADL was assessed using the Government-Certified Disability Index (GCDI) [12], which was developed for Japan’s Long-Term Care Insurance system. The GCDI is a five-level evaluation of physical and mental disorders requiring assistance in some or all ADLs. Nutritional status was assessed by dietitians using the Short-Form Mini-Nutritional Assessment (MNA-SF) [13]. Physiotherapists assessed cognitive functions using the ABC Dementia Scale [14]. The ABC Dementia Scale has a total score of 117–101 for normal or suspected dementia, 100–86 for mild, 85–71 for moderate, and 70–13 for severe dementia [15]. On the survey day, a dentist collected data on the state of the tooth defect, which was evaluated using the Eichner Index [16]. This indicator classifies occlusal states into 10 groups based on the occlusal support provided by the remaining natural teeth. When the participants wore dentures regularly, they were evaluated with the dentures on.

### 2.5. Statistical Analysis

The following were statistically compared between the group with a normal diet or a slightly soft diet (the normal diet group) and the group with a finely minced soft diet covered with a thick sauce or made into a mousse (the dysphagia diet group). Age, BMI, the ABC Dementia Scale, and the results of all masticatory kinematic analyses were compared using the independent *t*-test. The GCDI, MNA-SF, and Eichner Index were compared using the Mann–Whitney *U* test. In contrast, sex and cerebrovascular disease history were compared using the chi-square test. Additionally, simple linear regression analysis was performed to examine the relationship between the research items with significant differences and the intake of dysphagia diets. To determine the factors useful for predicting the need for a dysphagia diet, logistic regression analysis was performed using the intake of dysphagia diets as the objective variable and the items that showed significant differences in the comparison between the two groups as explanatory variables. The level of significance was set at *p* < 0.05. A receiver operating characteristic (ROC) curve was used to identify patients consuming a dysphagia diet. The area under the curve (AUC) and test accuracy were calculated. All statistical analyses were performed using SPSS (IBM SPSS Statistics for Windows Version 28.0, IBM Japan Ltd., Tokyo, Japan).

### 2.6. Sample Size Calculation

In a previous study, the tongue pressures of the normal and dysphagia diet groups were 17.7 ± 4.5 kPa and 13.2 ± 6.3 kPa, respectively (d = 0.76) [17]. The sample size was calculated assuming that the kinematic analysis of mastication differed as significantly as the tongue pressure. As a result of the calculation using G * Power (ver. 3.1.9.4, Universität Kiel, Kiel, Germany) with an effect size of 0.76, α of 0.05, and power (1-β) of 0.8, a minimum sample size of 58 participants was required.

## 3. Results

### 3.1. Characteristics of the Participants

**Figure 2:**
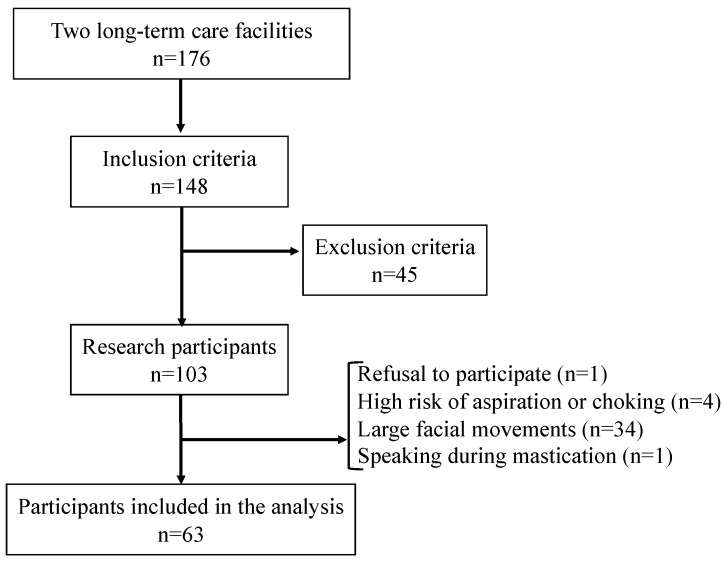
Flow chart of the participants.

The number of residents in both facilities was 176, and 148 participants met the inclusion criteria. Forty-five participants were excluded, and 103 underwent the measurements. However, one refused to participate in the experiment and was excluded. At the time of the investigation, three participants were unable to chew a baby rice cracker properly due to poor wakefulness, and one had a fever. Therefore, they were excluded based on the judgment that they were at high risk of aspiration or choking on rice crackers. Furthermore, 34 participants who made large facial movements during mastication and one who spoke continuously during mastication were also excluded. Thus, 38.8% of those who met the recruitment criteria were not suitable for kinematic analysis. Therefore, analysis was performed using recorded images of 63 participants (15 males, mean [SD] age: 88.1 [6.1] years) (Figure 2).

The normal diet group included 49 participants (11 men, mean [SD] age: 87.6 [6.5] years) and 14 in the dysphagia diet group (four men, mean [SD] age: 89.6 [4.1] years). In the normal diet group, 16 participants had a history of stroke, and one had a history of Parkinson’s disease. In the dysphagia diet group, four had a history of stroke, and one had a history of Parkinson’s disease. No disease that affected oral function other than dementia occurred in the participants. Regarding GCDI, the normal diet group received less care than did the dysphagia diet group; however, the groups did not significantly differ. Regarding nutritional status, there was no significant difference in MNA-SF; however, BMI was significantly lower in the dysphagia diet group than in the normal diet group (*p* < 0.001). The dysphagia diet group had significantly lower cognitive function than the normal diet group (*p* = 0.043). There was no significant difference in the Eichner Index between the groups (Table 1).

### 3.2. Kinematic Analysis Results

**Figure 3:**
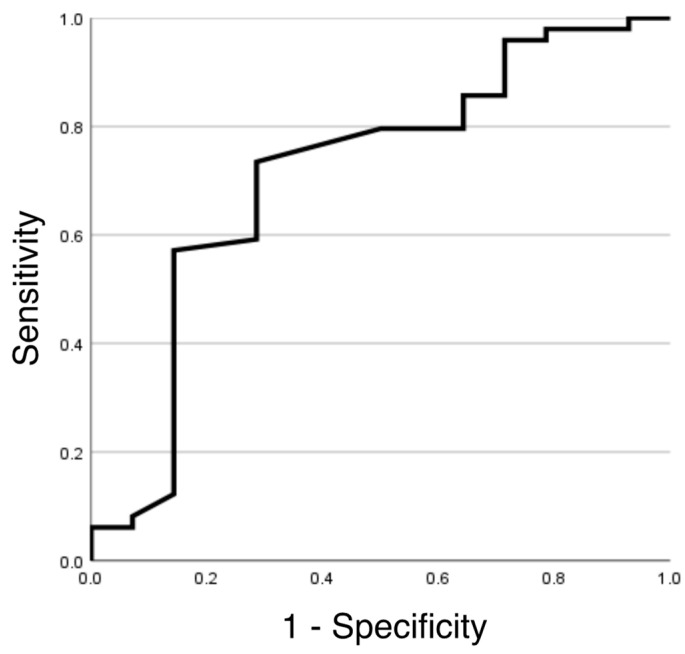
Receiver operating characteristic curves to identify patients taking a dysphagia diet by circular motion frequency.

When the values obtained by analyzing the masticatory motion were compared between both groups, the dysphagia diet group had significantly higher masticatory time, total change amount, and number of linear motions than those of the normal diet group. In addition, the cycle frequency and the circular motion frequency were significantly lower in the dysphagia diet group than in the normal diet group. However, no significant difference was observed in the number of cycles, number of circular motions, average speed, maximum speed, or coefficient of variation of the speed (Table 2). Additionally, simple linear regression analysis was used to examine the relationship between the research items with significant differences and the intake of dysphagia diets, revealing that masticatory time, total change amount, and circular motion frequency were significantly different (Table 3). Based on these findings, a logistic regression analysis was conducted using masticatory time, total change amount, and circular motion frequency as the explanatory variables. There was a significant difference in the circular motion frequency, and the odds ratio was −0.307 (Table 4). The cut-off value of the circular motion frequency calculated from the ROC curve was 63%, with a sensitivity of 0.714, a specificity of 0.735, and an AUC of 0.714 (Figure 3).

## 4. Discussion

In order to develop a screening test that can determine whether it is necessary to provide a dysphagia diet, the masticatory movements associated with the chewing of rice crackers in geriatric individuals in long-term care facilities were kinematically analyzed. The analyzed results were compared between the dysphagia diet group and the normal diet group. Significant differences were observed in the masticatory time, the cycle frequency, the total change amount, the number of linear motions, and the circular motion frequency. Regarding the effect size, masticatory time and total change amount were relatively large, and linear motion and circular motion frequency were moderate. Therefore, these differences in kinematic data during mastication are considered to be characteristics of masticatory movements in the elderly who are provided with a dysphagia diet. In particular, masticatory time and total change amount are considered remarkable features. Furthermore, results from multivariate analysis and AUC suggested that the circular motion frequency could be used as an indicator for the need to provide a dysphagia diet.

### 4.1. The Evaluation Method of Masticatory Movement

In this study, the same rice crackers used in a previous study were used for evaluation [10]. This rice cracker mixes easily with saliva when chewed and forms a bolus in oil; therefore, people with dysphagia have a relatively low risk of aspiration and choking [10]. Furthermore, we observed the chewing movement of the baby rice cracker and excluded participants at such risk to reduce the aspiration risk as much as possible. Therefore, we could prevent choking and aspiration. A previous study reported high inter-rater reliability for evaluating masticatory motion based on this mandibular movement [10]. Furthermore, in this study, the mandibular movement was classified into two types and measured, exhibiting high intra-rater and inter-rater reliability. Therefore, this test can be considered a relatively safe and reliable test for masticatory motion, even in participants with impaired cognitive function and masticatory ability. Compared to testing methods that use gummy jelly or gum, the merits of this test are that it can be applied to subjects who cannot keep their promise to spit it out after chewing and that the cost of the ingredients for testing is overwhelmingly low. However, the disadvantage is that it is not possible to check whether the bolus is sufficiently formed. Therefore, this test is recommended for use as a screening test for chewing ability.

### 4.2. The Kinematic Differences in Masticatory Movement

The timing from chewing to swallowing differs depending on the food type, but there is little individual variation in the particle size distribution of the boluses before swallowing [18]. If the particle size distribution of the bolus before swallowing did not differ significantly between the groups in this study, the results of increased masticatory time and total change amount suggested that their masticatory efficiency was relatively low. When observing masticatory movements, we noticed that some participants in the dysphagia diet group occasionally exhibited characteristic movements. For example, small movements, such as hesitating to chew, or unnecessary rest time between the end of one cycle and the start of the next. These unnecessary movements may have reduced the cycle frequency. Furthermore, the circular motion frequency was relatively low in the dysphagia diet group. In addition, multivariate analysis showed that the circular motion frequency was most closely related to the provision of a dysphagia diet. Kato et al. reported that the number of teeth and masticatory movement patterns were related to the ability to eat a normal diet [19]. The tear-shaped or oval masticatory pathway and the linear pathway are reportedly associated with good and low masticatory capacities, respectively [20]. In addition, a previous study suggested that masticatory pathways demonstrate a linear pattern in participants with inadequate bolus formation and that circular or elliptical masticatory pathways are essential for sufficient bolus formation [10]. Therefore, a decrease in circular motion during mastication could indicate reduced masticatory ability. In general, an AUC of 0.9 or more indicates high accuracy, 0.7 to less than 0.9 indicates moderate accuracy, and less than 0.7 indicates low accuracy [21]. Because the AUC of the circular motion frequency was greater than 0.7 in the results of this study, it could be used as a screening test to detect care recipients who need to be provided with a dysphagia diet. In other words, if the circular motion frequency was less than 63% when observing institutionalized residents chewing rice crackers, it indicates that their normal diet intake may be at risk.

In Japan, the percentages of residents with swallowing difficulties were reported to be 31.8% in nursing homes and 63.0% in long-term care facilities [22]. A resident’s masticatory ability gradually declines because of several factors [3,4,5]. However, because the changes are not abrupt, caregivers may not notice them. Using the results of this study, it is possible that the risk of aspiration and suffocation can be reduced by providing opportunities for caregivers to notice these changes and review their diet.

### 4.3. Consideration of Factors Affecting Masticatory Movement

This study could not identify the cause of the differences in kinematic data during mastication between the groups with different dietary forms. However, there was a significant difference in cognitive function but not in the Eichner index. This may be due to decreased motor function rather than the oral environment. Many reports suggest that cognitive decline reduces masticatory efficiency [23]. In addition, a study in mice also suggested that a reduction in masticatory afferent stimulation from long-term soft diet feeding could induce neuronal loss in the hippocampus and a decline in memory/learning ability [24]. Considering that masticatory function and cognitive function are closely related, the differences in the cerebral activity of the participants may have influenced the results of this study. On the other hand, it has been reported that masticatory ability is also affected by sarcopenia [4]. Among the participants in this study, the dysphagia diet group had a significantly lower BMI than the normal diet group, but it is unclear whether the proportion of participants with sarcopenia was skewed between the two groups. In addition, we did not measure the strength of mastication-related muscles. Since people requiring nursing care often have multiple factors that adversely affect masticatory function, it is important to understand the relationship between these factors and masticatory movement. Further investigations in more diverse populations are needed to reveal generalizable findings on how each of the muscles, oral environment, and brain activity influences masticatory movements.

### 4.4. Limitations

There were several limitations to this study. First, only one test food was used, and it is unclear whether other foods would show similar results. Furthermore, the test was conducted once; therefore, the reproducibility of the method could not be verified. Second, food processing and stage II transport progress during mastication [25]. In other words, it should be noted that the mandibular movements observed during this time reflect both the movements for food processing and stage II transport in the analysis results. Third, bolus properties were not observed. Therefore, we were unable to confirm the ability of participants to form a sufficient bolus before swallowing. Finally, this study did not examine the brain activity of the participants. The complex control of chewing and swallowing involves multiple cortical and subcortical regions, including limbic and prefrontal regions of the cortex [26]. Therefore, investigating brain activity is crucial to understanding our results. We will continue to investigate the factors that change the masticatory movements of facility residents.

## 5. Conclusions

In this study, we analyzed the mandibular movement during the mastication of rice crackers. The dysphagia diet group was associated with a decrease in cycle frequency and circular motion frequency, an increase in masticatory time, total change amount, and the number of linear motions compared to the corresponding values in the normal diet group. Moreover, we suggest that circular motion frequency could be used as a screening test to identify people who need to be provided with a dysphagia diet.

## Figures and Tables

**Table 1:** Characteristics and comparison of two groups divided by diet form.

	Total	Diet Form		*p* Value
	(n = 63)	Normal (n = 49)	Dysphagia (n = 14)	
Age (years) *	88.1 (6.1)	87.6 (6.5)	89.6 (4.1)	0.278 ^†^
Sex: male/female (n)	15/48	11/38	4/10	0.635 ^§^
GCDI (n)				0.116 ^‡^
1/2/3/4/5	9/8/19/20/7	9/5/17/13/5	0/3/2/7/2	
Cerebrovascular disease (n)	20	16	4	0.722 ^§^
BMI (kg/m^2^) *	21.03 (3.05)	21.69 (3.00)	18.73 (2.24)	<0.001 ^†^
MNA-SF				0.444 ^‡^
Normal (n)	8	8	0	
At risk (n)	48	35	13	
Malnutrition (n)	7	6	1	
ABC Dementia Scale *	77.0 (19.8)	79.7 (19.6)	67.6 (18.2)	0.043 ^†^
Eichner Index (n)				0.786 ^‡^
A1/A2/A3/B1/B2/B3	47/0/1/4/9/2	37/0/1/2/8/1	10/0/0/2/1/1	

GCDI: Government-Certified Disability Index; MNA-SF: Short-Form Mini-Nutritional Assessment; * presented as the mean (SD). ^†^: Independent *t*-test; ^‡^: Mann–Whitney *U*-test; ^§^: chi-square test.

**Table 2:** Comparison of kinematic analysis results.

	Diet Form		*p* Value	d	1-β
	Normal (n = 49)	Dysphagia (n = 14)			
Masticatory time (s)	28.90 (12.19)	38.95 (17.7)	0.027	0.731	0.660
Number of cycles (n)	37.15 (14.84)	42.20 (20.78)	0.241	0.306	0.168
Cycle frequency (n/s)	1.36 (0.37)	1.13 (0.35)	0.015	0.645	0.554
Total change amount (mm)	790.41 (300.89)	1061.72 (446.15)	0.024	0.805	0.530
Speed (mm/s)					
Average	29.32 (9.49)	29.70 (13.43)	0.922	0.036	0.052
Maximum	142.08 (54.88)	140.26 (56.51)	0.914	0.033	0.051
Coefficient of variation	0.79 (0.10)	0.80 (0.11)	0.799	0.077	0.057
Cycle types					
Circular motion (n)	24.93 (10.72)	24.10 (14.77)	0.787	0.070	0.056
Linear motion (n)	12.20 (9.98)	18.10 (13.61)	0.041	0.537	0.415
Circular motion frequency (%)	67.34 (16.10)	56.75 (21.89)	0.023	0.598	0.492

Presented as the mean (standard deviation).

**Table 3:** Univariable analysis.

	Univariable Analysis			
Variables	Odds Ratio	95% Confidence Interval	SE	*p* Value
Masticatory time	0.347	0.004–0.021	0.004	0.005
Cycle frequency	−0.243	−0.604–0.006	0.153	0.055
Total change amount	0.256	0.000–0.001	0.000	0.043
Circular motion frequency	−0.343	−0.014–0.002	0.003	0.006

**Table 4:** Multivariable analysis.

	Multivariable Analysis			
Variables	Odds Ratio	95% Confidence Interval	SE	*p* Value
Masticatory time	0.083	−0.009–0.015	0.003	0.631
Cycle frequency	−0.196	−0.585–0.102	0.006	0.165
Total change amount	0.213	0.000–0.001	0.172	0.171
Circular motion frequency	−0.307	−0.013–−0.002	0.000	0.011

## Data Availability

The data that support the findings of this study are available on request from the corresponding author. The data are not publicly available due to privacy or ethical restrictions.
